# SARS-CoV-2 testing, infection and places of contamination in France, a national cross-sectional study, December 2021

**DOI:** 10.1186/s12879-023-08257-1

**Published:** 2023-05-03

**Authors:** Sophie Vaux, Arnaud Gautier, Noemie Soullier, Daniel Levy-Bruhl

**Affiliations:** Santé publique France, The National Public Health Agency, 12, rue du Val d’Osne, Saint Maurice, 94415 France

**Keywords:** COVID-19, SARS-CoV-2 testing, Places of contamination, Infection, General population, Healthcare professionals

## Abstract

**Background:**

This study aimed to describe the use of diagnostic testing for SARS-CoV-2 in France until December 2021, the characteristics of people infected, and places of contamination.

**Methods:**

Data were collected from the national 2021 Health Barometer cross-sectional study, which was conducted between February and December 2021 and included French-speaking individuals aged 18–85 years old selected through randomly generated landline and mobile phone numbers. Participants were interviewed about COVID-19-like symptoms in the previous 12 months, diagnostic testing for SARS-CoV-2, positive diagnosis for SARS-CoV-2, and the place(s) of contamination. Determinants of diagnostic testing and of infection were studied using univariate and multivariate Poisson regressions.

**Results:**

A total of 24,514 persons participated in the study. We estimated that 66.4% [65.0-67.7] of persons had been tested for SARS-CoV-2 the last time they experienced COVID-19-like symptoms, and that 9.8% [9.3–10.3] of the population in France - with or without symptoms - had been tested positive. Diagnostic testing was less frequent in men, unemployed persons, and people living alone; it was also less frequent during the first months of the pandemic. The estimated proportion of the population infected was higher in healthcare professionals (PRa: 1.5 [1.3–1.7]), those living in large cities ( > = 200 000 inhabitants, and Paris area) (1.4 [1.2–1.6]), and in households comprising > 3 persons (1.7 [1.5-2.0]). It was lower in retired persons (0.8 [0.6–0.97]) and those over 65 years old (0.6 [0.4–0.9]). Almost two-thirds (65.7%) of infected persons declared they knew where they were contaminated; 5.8% [4.5–7.4] reported being contaminated outdoors, 47.9% [44.8–51.0] in unventilated indoor environments, and 43.4% [40.3–46.6] in ventilated indoor environments. Specifically, 51.1% [48.0-54.2] declared they were contaminated at home or in a family of friend’s house, 29.1% [26.4–31.9] at their workplace, 13.9% [11.9–16.1] in a healthcare structure, and 9.0% [7.4–10.8] in a public eating place (e.g., cafeteria, bar, restaurant).

**Conclusions:**

To limit viral spread, preventive actions should preferentially target persons tested least frequently and those at a higher risk of infection. They should also target contamination in households, healthcare structures, and public eating places. Importantly, contamination is most frequent in places where prevention measures are most difficult to implement.

**Supplementary Information:**

The online version contains supplementary material available at 10.1186/s12879-023-08257-1.

## Background

Almost three years after the identification of the first cases of coronavirus disease 2019 (COVID-19) in Wuhan, China [1], the associated pandemic continues.

The first cases in France were detected at the end of January 2020 [2]. The first wave occurred in March-April 2020. The first national lockdown was implemented in mid-March 2020. Between October and November 2020, a second wave occurred. New social distancing measures were put in place including curfews, self-isolation, teleworking, and school closures. The Alpha variant emerged in France at the beginning of 2021. Its incidence increased until April 2021 and remained very high until May 2021, constituting the third COVID-19 wave. The Delta variant emerged in June 2021, at a time when overall SARS-COV-2 (the virus responsible for COVID-19) incidence was decreasing and public places (restaurants, bars, outdoor terraces, and cultural sites) were being reopened. Its circulation increased during summer 2021 constituting the fourth wave. It gave way to the Omicron variant in December 2021 which peaked in January 2022.

The COVID-19 vaccination campaign started in France on December 27, 2020. The vaccines were recommended at first to those most likely to develop a severe COVID-19 disease (people aged ≥ 75 years old, residents of nursing homes and long-term care facilities, people with chronic conditions, and healthcare workers). In February 19, 2021, the campaign opened up to people aged 50 years and older with comorbidities. The campaign was extended to all adults by mid-May 2021 and to children ≥ 12 years old in mid-June 2021.

The COVID-19 epidemiological situation is constantly monitored in France by Santé publique France, the French national public health agency, which publishes daily updates [3]. Epidemiological surveillance includes the description and evolution of the number of persons tested for SARS-CoV-2, of persons testing positive for SARS-CoV-2, hospitalizations, and related deaths.

SARS-CoV-2 is transmitted through close contact, aerosols, droplets and contaminated surfaces. Transmission has been described in many places including restaurants and bars [4], nursing homes [5], healthcare facilities [6], buses [7], cruise ships [8] and airplanes [9]. In order to better understand the places and activities facilitating transmission, case-control studies have been conducted in France [10, 11].

The main objectives of the present study were to describe the use of diagnostic testing for SARS-CoV-2 in France until December 2021, the characteristics of people infected, and places of contamination.

## Methods

### Study design

We used data collected from the 2021 French Health Barometer of Sante publique France. This national cross-sectional survey investigates health-seeking behaviours and perceptions, and has been regularly conducted by Santé publique France since 1992 [12]. The sampling method is based on random digit dialling of landline and cellular telephone numbers. Participants are selected according to a two-stage sampling design for landline phone numbers (selection of one individual per household according to the Kish method [13]), and a one-stage sampling design for cellular numbers (the person who picks up is selected). Each generated number is called up to 40 times at various times of the day and week in order to include individuals with limited availability.

### Data collection tool

Participants are interviewed using the Computer Assisted Telephone Interviewing [14] system, in which the interviewer conducts the interview over the telephone while following a pre-established script on a personal computer screen. Only interviewees who are fluent in French are included. The survey method has been described elsewhere [15]. The questions were validated during a pilot study including 179 participants. The questionnaire is available online [16].

### Sample size

The French Health Barometer of Sante publique France is a multi-thematic survey including questions on many subjects. The sample size was calculated to have at least 1000 participants in each the 12 regions of metropolitan France (the Provence-Alpes Côte d’Azur and Corsica regions were combined into one region), allowing sufficient precision for regional estimates of the main variables of interest (coefficient of variation < 10%).

### Data collection

Data were collected from 11 February to 30 December 2021 (with a summer break from 19 July to 22 August 2021). The questionnaire included 14 questions on COVID-19 symptoms, diagnostic testing for SARS-CoV-2, results, and place of contamination.

Participants were asked about the presence of COVID-19-like symptoms in the previous 12 months (“In the past 12 months, have you had any symptoms or signs of illness that made you think you might have had COVID-19?”). Those who replied “yes” were asked when (month, year) their most recent symptoms occurred. All participants (i.e., whether reporting symptoms or not) were asked if they had undergone at least one diagnostic test for SARS-CoV-2 (including PCR tests, rapid antigen tests with self and professional performances”, but excluding serological tests) in the 12 months. Those who replied “yes” were then asked the result of their test. If it was positive, participants were asked if they knew where they had been contaminated (1.Yes, you are sure, 2.Yes, but you are not entirely sure, 3.No, not really, 4. Don’t know, 5. Refusal to answer). Participants who declared they knew the place of contamination were then asked to describe it (1. Place of study, 2. Place of work, 3. Own, family or friends’ home, 4. During a sporting activity, 5. Place of worship, 6. Shopping, in a shop, 8. During a cultural activity (theatre, cinema, library), 9. Public transport, 10. Healthcare structure (hospital, medical or paramedical practice, laboratory), 11. Restaurant or bar, 12. Other, 13. Refusal to answer), and to specify the ventilation conditions (1. Outside, 2. Unventilated inside (windows closed), 3. Inside ventilated (windows open), 4. Don’t know, 5. Refusal to answer).

Other information was collected: for participants: gender, age, professional situation, education level, city of residence, nationality, declaration of a chronic disease and for the household: size, number and age of children, income per month (euros).

### Data analysis

The determinants investigated for (i) the proportion of persons with symptoms tested for SARS-CoV-2, (ii) those diagnosed SARS-CoV-2 positive within the previous 12 months (whether symptomatic or asymptomatic), and (iii) for the various places of contamination were as follows: gender, age, professional situation (general population: employed, student, unemployed, retired, other inactive not looking for a job (for instance homemakers), size of household (classified into 1, 2, 3, and 4 or more persons), number of children under 14 in household, having children under 5 years old, education level (lower secondary education and under, upper secondary school certificate, two years higher education, three/four years higher education, five years or more higher education), being a healthcare professional, income per month (euros) per household consumption unit in tertiles (< 1170, 1170 to 1800, > 1800), region of residence, urban unit size (rural, < 20 000 inhabitants, 20 000–99 999, 100 000-199 999, >=200 000 and Paris area), nationality (French nationality by birth, French nationality acquired, non-French nationality), chronic disease. For the analysis of the proportion of symptomatic persons tested for SARS-CoV-2, the time of symptom onset was recorded as one of five time periods: January-May 2020, June-July 2020, August 2020-February 2021, March-August 2021, September-December 2021. These time periods were chosen according to availability of diagnostic tests and to the epidemiological situation in France. For the proportion of persons (symptomatic or asymptomatic) with at least one positive SARS-CoV-2 test in the previous 12 months, the time of the interview was classified as one of the following four time periods: January-March 2021, April-June 2021, July-September 2021 and October-December 2021. Analyses were performed using univariate and multivariate Poisson regressions.

Prevalence ratios (PR) and adjusted prevalence ratios (PRa) were used to measure associations. Determinants with a p-value < 0.2 in the univariate analyses were included in the multivariate analyses and presented in the tables. The final multivariate analysis was constructed using backward elimination and only variables with a p-value < 0.05 were retained. In order to test for possible confounding effects, each of the variables excluded from the final model were reintroduced individually and tested for significance. Confounding was determined if a change of ≥ 30% in the regression coefficients was observed. Collinearity and interaction were tested when relevant. Due to interactions between health professionals and gender for persons reporting diagnostic testing, stratified analyses were performed separately for health professionals and the general population (i.e., excluding health professionals).

All analyses were performed using Stata SE.64 15.1 (StataCorp, USA). Estimates were weighted to take into account (i) the selection probability (i.e., within the household and according to the type of telephone call (landline versus cellular)), and (ii) the structure of the French population. Specifically, calibration weighting was used with the following covariates: gender crossed with age in ten-year groups and region of residence, size of urban unit, size of household, and education level (reference population: French National Institute for Statistics and Economic Studies (Insee), 2020 Labor Force Survey). All analyses were performed with the ‘svy’ command, which takes into account the weights in all the estimates (i.e., descriptive analyses, confidence intervals, Poisson regression). The results are presented with unweighted numbers, percentages and their confidence intervals (95% CI). Chi2 Pearson tests (designed-based correction) were calculated to compare percentages.

## Results

### Participants

A total of 24,514 participants were included (17,496 interviewed on cellular phones (71%) and 7,018 on landlines (29%)). The participation rate was 44%.

### Persons with COVID-19-like symptoms tested for the disease and determinants

We estimated that 21.7% [21.1–22.4] and 10.2% [9.7–10.6] of the population in France presented COVID-19-like symptoms once and more than once, respectively, in the previous 12 months; 68.0% [67.3–68.8] reported not having any such symptoms (0.03% [0.008-0.1] and 0.08% [0.04–0.2], respectively, refused to answer and did not know).

Two-thirds of persons reporting symptoms (66.4% [65.0-67.7]) reported diagnostic testing (excluding serology) the last time they were symptomatic. One-third (35.5% [33.8–37.2]) of those tested had a positive diagnosis over the entire study period.

In asymptomatic people, 55.5% [54.6–56.5] reported they had been tested (again, excluding serology). Of those tested, 4.0% [3.6–4.6] reported a positive diagnosis.

In the general population (i.e., healthcare professionals excluded), in terms of symptomatic persons, the multivariate analyses highlighted that women were more frequently tested than men, that unemployed persons were less frequently tested than employed persons, and that individuals living alone were less frequently tested (59.6% [56.4–62.7]) than those living in 2-, 3-, and ≥ 4-person households. The proportion of symptomatic individuals tested for SARS-CoV-2 varied according to the symptom-onset period: the proportion was lower for January-May 2020, June-July 2020, and September-December 2021 (vs. August 2020-February 2021, taken as reference (Table 1).

**Table 1:** Proportions of symptomatic persons tested for SARS-CoV-2 and determinants, general population (healthcare professionals excluded), Baromètre de Santé publique France, 2021

	Proportion of persons tested for SARS-CoV-2				
	** N**	**% [CI95%]**	**PR** [**CI95%]**	**PRa** [**CI95%]**	**p-value**
	**6932**	**65.6 [64.2–67.0]**			
**Gender**					
Man	3305	63.1 [61.0-65.2]	Reference	Reference	
Woman	3627	**67.9 [66.0–70.0]**	**1.1 [1.0-1.1]**	**1.1 [1.0-1.1]**	**0.002**
**Professional situation**					
Employed	4429	67.0 [65.2–68.8]	Reference	Reference	
Student	665	69.9 [65.7–73.8]	1.0 [1.0-1.1]	1.0 [1.0-1.1]	0.9
Unemployed	483	**58.7 [53.2–63.9]**	**0.9 [0.8–0.96]**	**0.9 [0.8–0.96]**	**0.006**
Retired	1074	61.7 [58.0-65.3]	0.9 [0.9–0.98]	1.0 [0.9-1.0]	0.3
Other inactive	281	62.4 [55.2–69.1]	0.9 [0.8-1.0]	0.9 [0.8-1.0]	0.06
**Household size (persons)**					
1	1461	59.6 [56.4–62.7]	Reference	Reference	
2	2162	**66.1 [63.6–68.5]**	**1.1 [1.0-1.2]**	**1.1 [1.0-1.2]**	**0.003**
3	1239	**65.9 [62.6–68.5]**	**1.1 [1.0-1.2]**	**1.1 [1.0-1.1]**	**0.046**
4 or more	2070	**68.0 [65.5–69.1]**	**1.1 [1.1–1.2]**	**1.1 [1.1–1.2]**	**0.03**
**Period of symptom onset**					
January - May 2020	1259	**31.0 [27.9–34.2]**	**0.7 [0.6–0.8]**	**0.7 [0.7–0.9]**	**< 0.001**
June - July 2020	2940	**55.1 [47.8–62.1]**	**0.4 [0.4–0.5]**	**0.4 [0.4–0.5]**	**< 0.001**
August 2020 - February 2021	1259	74.6 [72.6–76.6]	Reference	Reference	
March - August 2021	1932	75.5 [73.1–77.8]	0.8 [0.7–0.9]	1.0 [1.0–1.0]	0.8
September - December 2021	485	**58.9 [53.3–77.8]**	**0.8 [0.5–1.1]**	**0.8 [0.7–0.9]**	**< 0.001**
Don’t know	32	58.5 [38.1–76.3]	0.7 [0.7–0.8]	0.8 [0.6–1.2]	0.3

**PRa are adjusted for region**: persons residing in the Nouvelle Aquitaine (62.4% [57.0-67.5], PRa = 0.9 [0.8–0.99], p = 0.03) and Auvergne Rhone Alpes (63.2% [59.3–66.8], PRa = 0.9 [0.9–0.99], p = 0.02) regions were less frequently tested than those residing in the Ile-de-France region (reference) (66.4% [63.4–69.4]).

Symptomatic healthcare professionals were more frequently tested for SARS-CoV-2 than the general population (74.0% [69.5–78.1] vs. 65.6% [64.2–67.0], p < 0.001). Among them, people in the ‘other inactive’ subgroup (excluding unemployed and retired healthcare professionals) were less frequently tested than employed professionals, and those living alone were more frequently tested than those living in a 2-person household. The proportion of tested symptomatic professionals was lower for January-May 2020 and June-July 2020 (vs. August 2020-February 2021, taken as reference) (Table 2).

**Table 2:** Proportions of symptomatic persons tested for SARS-CoV-2 and determinants, healthcare professionals, Baromètre de Santé publique France 2021

	Proportion of healthcare professionals tested for SARS-CoV-2				
	** N**	**% [CI95%]**	**RP [CI95%]**	**RPa [CI95%]**	p
**All healthcare professionals**	735	74.0 [69.5–78.1]			
**Professional situation**					
Employed	525	80.3 [75.5–84.4]	Reference	Reference	
Student	29	76.2 [56.1–88.8]	0.9 [0.8–1.2]	0.9 [0.7–1.1]	0.2
Unemployed	30	65.8 [44.8–81.9]	0.8 [0.6–1.1]	0.8 [0.6–1.1]	0.1
Retired	119	61.1 [48.6–72.4]	0.8 [0.6–0.9]	0.8 [0.7-1.0]	0.1
Other inactive	32	**39.2 [20.1–62.3]**	**0.5 [0.3–0.9]**	**0.5 [0.3–0.8]**	**0.005**
**Household size**					
1	135	79.2 [70.0-86.3]	Reference	Reference	
2	258	**65.9 [57.7–73.2]**	**0.8 [0.7–0.97]**	**0.8 [0.7–0.9]**	**0.003**
3	126	80.0 [69.6–87.4]	1.0 [0.9–1.2]	0.9 [0.7-1.0]	0.07
4 or more	216	76.2 [67.5–83.2]	1.0 [0.8–1.1]	0.9 [0.8-1.0]	0.08
**Education level**					
Lower secondary education and under	151	64.4 [54.2–73.5]	Reference		
Upper secondary school certificate	111	77.2 [65.1–84.9]	1.2 [1.0-1.4]		
Two years higher education	58	79.9 [65.1–89.4]	1.2 [1.0-1.5]		
Three/four years higher education	245	81.4 [75.6–86.0]	1.3 [1.1–1.5]		
Five years or more higher education	164	77.8 [69.6–84.3]	1.2 [1.0-1.4]		
**Period of symptom onset**					
January - May 2020	137	**43.6 [33.4–54.4]**	**0.5 [0.4–0.6]**	**0.5 [0.4–0.6]**	**< 0.001**
June - July 2020	27	**50.4 [29.0-71.6]**	**0.6 [0.4–0.9]**	**0.6 [0.4–0.9]**	**0.02**
August 2020 - February 2021	319	86.7 [81.2–90.8]	Reference	Reference	
March - August 2021	181	79.9 [71.5–86.4]	0.8 [0.7–1.1]	0.9 [0.8-1.0]	0.2
September - December 2021	67	73.2 [55.1–85.9]	1.2 [1.1–1.2]	1.1 [0.7-1.0]	0.09
Don’t know	4	-			

*PRa are adjusted for region*: no significant difference was found between regions (Ile-de-France region taken as reference) (67.7% [54.7–78.5]).

### Proportion of persons with at least one positive COVID-19 diagnosis in the previous 12 months and related determinants

Over the whole study period, we estimate that 9.8% [9.3–10.3] of the population in France was infected (i.e., tested positive) with COVID-19 at least once in the previous 12 months, whether symptomatic or not.

According to our multivariate analysis, the following sub-groups were more likely to test positive: healthcare professionals (vs. those who are not), persons living in large cities including 100 000-199 999 inhabitants, >=200 000 inhabitants and the Paris area (vs. rural areas), persons living in households with more than one person (vs. single-person households).

Retired persons and those in the ‘other inactive’ sub-group were less likely to have tested positive (vs. those employed) and those older than 65 years old in comparison with those aged 18–24 years. The proportion of persons with at least one positive SARS-CoV-2 test in the previous 12 months was higher for persons interviewed in the most recent time period (Table 3).

**Table 3:** Proportion of persons with at least one positive SARS-CoV-2 test and determinants, general population, Baromètre de Santé publique France, 2021

	Proportion of persons with at least one positive SARS-CoV-2 test in the previous 12 months				
	N	**% [CI95%]**	**PR [CI95%]**	**PRa [CI95%]**	**p-value**
	**24 514**	**9.8 [9.3–10.3]**			
**Healthcare Professional**					
No	22 398	9.4 [8.0–10.0]	Reference	Reference	
Yes	2 116	14.1 [12.2–16.3]	**1.5 [1.3–1.7]**	**1.5 [1.3–1.7]**	**< 0.001**
**Professional situation**					
Employed	13 278	11.7 [11.0-12.4]	Reference	Reference	
Student	1 393	16.0 [13.9–18.4]	1.4 [1.2–1.6]	1.2 [1.0-1.5]	0.05
Unemployed	1 386	9.5 [7.7–11.6]	0.8 [0.7-1.0]	0.8 [0.7-1.0]	0.08
Retired	7 415	**5.3 [4.6–5.9]**	**0.4 [0.4–0.5]**	**0.8 [0.6–0.97]**	**0.03**
Other inactive	1 042	**8.1 [6.1–10.5]**	**0.7 [0.5–0.9]**	**0.7 [0.6–0.95]**	**0.02**
**Residential agglomeration size (inhabitants)**					
Rural	6 343	7.3 [6.6–8.2]	Reference	Reference	
<20 000	4 153	8.0 [7.1–9.2]	1.1 [0.9–1.3]	1.1 [0.9–1.3]	0.3
20 000–99 999	3 151	8.0 [6.9–9.3]	1.1 [0.9–1.3]	1.0 [0.9–1.3]	0.6
100 000–199 999	1 317	**9.4 [7.6–11.7]**	**1.3 [1.0-1.6]**	**1.3 [1.0-1.6]**	**0.045**
>=200 000 and Paris area	9 464	**12.6 [11.7–13.5]**	**1.7 [1.5–1.9]**	**1.4 [1.2–1.6]**	**< 0.001**
**Household size (persons)**					
1	6 063	6.7 [5.9–7.5]	Reference	Reference	
2	9 142	**8.5 [7.9–9.3]**	**1.3 [1.1–1.5]**	**1.4 [1.2–1.6]**	**< 0.001**
3	3 781	**9.8 [8.7–11.1]**	**1.5 [1.2–1.7]**	**1.2 [1.0-1.5]**	**0.02**
4 or more	5 528	**14.0 [12.8–15.2]**	**2.0 [1.8–2.4]**	**1.7 [1.5-2.0]**	**< 0.001**
**Age (years)**					
18–24	2 035	13.7 [12.0-15.6]	Reference	Reference	
25–34	3 220	13.1 [11.8–14.6]	1.0 [0.8–1.1]	1.1 [0.9–1.3]	0.5
35–44	3 851	11.8 [1.5–13.2]	0.9 [0.7-1.0]	0.9 [0.7–1.1]	0.5
45–54	4 530	10.2 [9.1–11.5]	0.7 [0.6–0.9]	0.9 [0.7–1.1]	0.2
55–64	4 662	8.9 [7.9–10.1]	0.6 [0.5–0.8]	0.9 [0.7–1.1]	0.4
65–85	6 216	**4.9 [4.3–5.6]**	**0.4 [0.3–0.4]**	**0.6 [0.4–0.9]**	**0.005**
**Chronic illness**					
Yes	10,326	8.6 [7.9–9.3]	Reference		
No	14,129	10.7 [10.0-11.4]	1.2 [1.1–1.4]		
**Nationality**					
French by birth	22 217	9.3 [8.9–9.8]	Reference		
French nationality acquired	1173	**13.3 [11.0-16.1]**	**1.4 [1.2–1.7]**	**1.2 [1.0-1.5]**	**< 0.05**
Non-French nationality	1100	13.3 [10.8–16.3]	1.4 [1.2–1.8]	1.1 [0.9–1.4]	0.3
Refusal to answer	24	14.7 [4.6–38.2]	1.6 [5.3–4.7]	1.9 [0.7–5.1]	0.2
**Number of household inhabitants under 14 years of age**					
0	18,856	8.7 [8.1–9.2]	Reference		
1	2800	12.6 [11.0-14.3]	1.4 [1.3–1.7]		
2	2213	11.9 [10.3–13.8]	1.4 [1.2–1.6]		
3 or more	645	17.0 [13.6–21.0]	2.0 [1.6–2.5]		
**Education level**					
Lower secondary education and under	7822	8.4 [7.6–9.2]	Reference		
Upper secondary school certificate	5091	10.8 [9.6–11.9]	1.3 [1.1–1.5]		
Two years higher education	3374	11.1 [9.8–12.5]	1.3 [1.1–1.5]		
Three/four years higher education	3906	12.0 [10.8–13.3]	1.4 [1.2–1.6]		
Five years or more higher education	4207	10.9 [9.8–12.1]	1.3 [1.1–1.5]		
**Family income per household; consumption units (i.e., euros per consumption unit)**					
< 1170	6649	10.2 [9.3–11.1]	Reference		
1170 to 1800	7477	9.0 [8.2–9.8]	0.9 [0.8–0.99]		
> 1800	8390	10.0 [9.1–10.8]	1.0 [0.9–1.1]		
**Period of study interview**					
February – March 2021	6078	6.4 [5.7–7.2]	Reference	Reference	
April – June 2021	7832	**9.1 [8.3–9.9]**	**1.4 [1.2–1.6]**	**1.4 [1.2–1.6]**	**< 0.001**
July – September 2021	4036	**10.9 [9.7–12.3]**	**1.7 [1.4-2.0]**	**1.6 [1.4–1.9]**	**< 0.001**
October – December 2021	6568	**12.8 [11.9–13.9]**	**2.0 [1.7–2.3]**	**1.8 [1.6–2.1]**	**< 0.001**

**PRa are adjusted for region**: persons living in the Auvergne Rhône Alpes region (12.8 [11.4–14.4], PRa = 1.3 [1.0-1.6], p = 0.02) were more frequently infected than those living in Hauts-de-France region (taken as reference) (9.9 [8.4–11.7]). Those living in the Brittany (4.6 [3.3–6.3], PRa = 0.5 [0.4–0.7], p < 0.001) and Nouvelle-Aquitaine (6.0 [4.9–7.4], PRa = 0.7 [0.5–0.9], p = 0.004) regions were less frequently infected.

### Places of contamination

Almost two-thirds (65.7% [61.2–70.4]) of persons who tested positive declared they knew where they had been contaminated (46.8% [44.2–49.5] were sure, 18.9% [17.0–20.9] were not completely sure), while 33.9% [31.4–36.5] said they did not know (refusal to answer: 0.1% [0.02–0.6]).

Asymptomatic persons who tested positive were less likely to report they knew where they were contaminated vs. symptomatic positive persons (knew location and were sure: asymptomatic 44.9% [38.5–51.5] vs. symptomatic 47.2% [44.4–50.0]; knew location but not sure: 11.8% [8.6–16.1] vs. 20.2% [18.1–22.5]; did not know location: 42.8% [36.2–49.5] vs. 32.2% [29.6–35.1], p = 0.005).

The principal place of contamination was in a home (51.1% [48.0-54.2] reported being contaminated in their own home or in a family or friend’s home). The determinants associated with contamination in a home in the multivariate analysis were as follows: (a) household size: those living in a household comprising at least 4 persons were more likely to report being contamination at home than those living alone (minimum 4-person household: 57.0% [51.5–62.3] vs. 1-person household: 42.3% [35.1–49.8], PRa:1.5 [1.2–1.8], p < 0.001); (b) professional situation : unemployed, retired and ‘other inactive’ persons were more likely to report being infected at home than employed persons (unemployed person: 60.6% [47.3–62.6 ] vs. employed person: 46.2% [42.4–50.1], PRa: 1.3 [1.0-1.6], p = 0.02; retired person: 59.3% [51.4–66.8], PRa: 1.4 [1.2–1.7], p < 0.001; ‘other inactive’ person: 71.6% [55.5–83.6], PRa:1.5 [1.2–1.8], p < 0.001).

The second and third most-reported places of contamination were, respectively, the workplace (29.1% [26.4–31.9]) and in a shop (5.4% [4.2–6.9]) (Table 4).

**Table 4:** Description of places of contamination, general population, Baromètre de Santé publique France, metropolitan France, 2021 (n: 1526)

		Outdoor	Unventilated indoor	Ventilated indoor	Refused to answer, Don’t know
Contamination places	% [CI95%]	% [CI95%]	% [CI95%]	% [CI95%]	% [CI95%]
All places	100.0	5.8 [4.5–7.4]	47.9 [44.8–51.0 ]	43.4 [40.3–46.6]	2.9 [2.1-4.0]
Own, family or friends’ home	51.1 [48.0-54.2]	4.3 [1.9–6.2]	44.3 [39.9–48.8]	47.7 [43.2–52.3]	3.7 [2.4–5.6]
Place of worship	29.1 [26.4–31.9]	3.5 [2.0–6.0]	53.2 [47.4–58.8]	41.2 [35.6–47.0]	2.2 [1.1–4.4]
In a shop, shopping	5.4 [4.2–6.9]	2.9 [0.5–15.6]	74.7 [61.1–84.8]	19.6 [10.7–33.0]	2.8 [0.8–9.6]
Healthcare structure (hospital, medical or paramedical practice, laboratory)	4.9 [3.8–6.2]		69.6 [54.4–81.4]	30.5 [18.6–45.6]	
Public transport	4.6 [3.6-6.0]	0.4 [0.05–2.9]	85.6 [72.7–92.9]	9.3 [3.7–21.3]	4.7 [1.3–16.2]
Restaurant, bar	3.8 [2.9-5.0]	16.4 [8.4–29.6]	46.0 [32.6–60.1]	36.3 [23.5–51.4]	1.3 [0.2–9.1]
Study place	2.8 [1.8-4.0]	8.8 [2.6–26.2]	36.7 [20.1–57.3]	53.0 [33.5–71.6]	1.5 [0.2–11.2]
Place of vacation, cottage, hotel, place of leisure	1.6 [1.0-2.7]	46.9 [23.1–72.2]	8.7 [2.6–25.3]	32.0 [11.7–62.4]	12.5 [3.6–35.3]
During a sporting activity	1.5 [1.0-2.4]	45.6 [23.4–69.7]	32.3 [14.3–57.8]	20.4 [7.0-46.3]	1.8 [0.2–13.5]
School, nursery, nanny	0.9 [0.5–1.9]				
Sports gathering, concert	0.8 [0.4–1.6]				
Wedding, funeral, family reunion	0.8 [0.4–1.8]				
Non-collective transport: car, ambulance, taxi	0.7 [0.3–1.4]				
Night club, club	0.7 [0.3–1.3]				
Place of worship	0.5 [0.2-1.0]				
Cultural activity (theatre, museum, library)	0.4 [0.2–0.8]				
Other place	0.1 [0.4–2.1]				

Many possible responses. The distribution according to ventilation characteristics is only indicated when the number of participants reporting was higher than 10

Among those reporting contamination at work, healthcare structures (hospital, medical or paramedical practice, laboratory) were most mentioned (31.7% [26.7–37.2]) (Table 5).

**Table 5:** Description of places of contamination in professional context, Baromètre de Santé publique France, metropolitan France, 2021 (n: 453)

		Outside	Unventilated indoor	Ventilated indoor	Refused to answer
Professional contamination places	% [CI95%]	% [CI95%]	% [CI95%]	% [CI95%]	% [CI95%]
**All professional places**	29.1 [26.4–31.9]	3.5 [2.0–6.0]	53.2 [47.4–58.8]	41.2 [35.6–47.0]	2.2 [1.1–4.4]
Healthcare structure (hospital, medical or paramedical practice, laboratory)	31.7 [26.7–37.2]	-	64.5 [54.0-73.7]	33.5 [24.5–44.0]	2.0 [0.6–6.6]
Office	23.1 [18.8–28.0]	-	63.8 [52.0–74.0]	35.7 [25.4–47.5]	0.6 [0.1–4.2]
Eating place (restaurant)	12.7 [9.4–17.0]	11.7 [4.5–27.4]	50.5 [34.5–66.4]	37.8 [23.5–54.7]	-
workshop, garage	8.0 [5.2–12.1]	2.3 [0.3–17.1]	37.6 [19.0-60.8]	56.7 [34.0-76.9]	3.4 [0.4–22.9]
Company cafeteria, coffee machine	6.3 [4.1–9.4]	1.9 [0.2–14.0]	33.6 [17.8–54.2]	64.5 [43.9–80.8]	-
School, nursery, recreation centre	5.9 [3.9–8.8]	-	8.5 [2.8–23.5]	86.4 [70.6–94.4]	5.1 [1.3–17.6]
Shop, market	4.7 [2.6–8.1]	-	66.0 [33.0-88.5]	34.0 [11.6–67.0]	-
Counter (customer reception area)	2.0 [1.0–4.0]	-	60.7 [22.3–89.3]	39.3 [10.7–77.7]	-
Seminar, congress	1.1 [0.4–2.8]				
Vehicle, bus, train station, plane	1.0 [0.5-2.0]				
Meeting	0.5 [0.2-1.0]				
Other place	2.3 [1.2–4.3]	39.1 [12.5–74.5]	41.4 [13.4–76.4]	15.5 [2.3–58.9]	4.1 [0.4–31.2]
Don’t know	3.0 [1.5–5.8]				

Various possible responses. The distribution according to ventilation characteristics is only indicated if the number of participants reporting was higher than 10

Healthcare structures, whether frequented in a working or patient/visitor context, accounted for 13.9% [11.9–16.1] of the all declared places of contamination.

Just under one-fifth (18.3% [14.4–23.1]) of persons who declared they knew where they were contaminated at work mentioned eating places (12.7% [9.4–17.0]), cafeteria or proximity to a coffee machine (6.3%[4.1–9.4]. Eating places (including restaurants, bars, cafeteria, coffee machine …), whether frequented in a working or customer context, accounted for 9.0% [7.4–10.8] of all the declared places of contamination.

### Indoor and outdoor contamination

Overall, 5.8% [4.5–7.4] of persons reported having been contaminated outdoors, 47.9% [44.8–51.0] in unventilated indoor environments, and 43.4% [40.3–46.6] in ventilated indoor environments (i.e., windows open); 2.9% [2.1-4.0] refused to answer or did not know (Table 4).

In terms of unventilated indoor environments, contamination was particularly prevalent in public transport (85.6% [72.7–92.9]) and in shops (74.7% [61.1–84.8]).

With regard to ventilated indoor environments, contamination was prevalent in homes (whether one’s own, a family or a friend’s) (47.7% [43.2–52.3]) and in a study place (53.0% [33.5–71.6]) (Table 4).

## Discussion

Only 66.4% [65.0-67.7] of persons who reported COVID-19-like symptoms had performed a diagnostic test.

The proportion of symptomatic persons tested for SARS-CoV-2 varied greatly over time. Between January and July 2020, it was low in the general population (31.0%) and in healthcare professionals (43.6%), most probably because of the lack of availability of diagnostic tests in France, especially early in this period. The tests were then mainly recommended for patients with severe symptoms and carried out in hospitals. The number of persons tested for SARS-CoV-2 in general population in France during this period was low (for instance: 380 persons tested/100 000 inhabitants mid-May 2020) [supplementary data, Fig. 1] [3]. The first pandemic wave occurred in France at this time (Wave 1: between the beginning of March and mid-May 2020, Weeks 11–19/2020). From August 2020 to August 2021, the proportion of persons tested was 75% or higher in both groups, in a context where diagnostic testing was widely available. During this period number of persons tested for SARS-CoV-2 increased from 1530/100 000 inhabitants to 6700/100 000 [3]. The second wave occurred between the beginning of October and the end November 2020 (Wave 2: Weeks 40–48/2020). Waves 3 and 4 were observed between the beginning of March and the end of May 2021 (Wave 3: Weeks 9–19/2021), and between mid-July and mid-October 2021 (Wave 4: Weeks W29-40/2021), respectively. COVID-19 incidence remained high throughout the period between Waves 3 and 4 [17] [Supplementary data, Figs. 1 and 2].

From September to December 2021, the proportion of symptomatic persons who performed a diagnostic test fell sharply (58.9%) in the general population. This decrease was not observed in symptomatic healthcare professionals (73.2%). Over this last period of the study, the Delta variant, followed by the Omicron variant, were dominant in France and were responsible for Waves 5 and 6. The Omicron wave that started in December 2021 was responsible for a very large number of cases. Less systematic testing over this period may be partly due to pandemic fatigue in the population in terms of testing, the lack of specific treatment for COVID-19, the lesser severity of the Omicron variant [18], and over-burdened places for screening (medical analysis laboratories, pharmacies, etc.) leading to difficulties in accessing tests. During the peak of the Omicron wave, the incidence rate reached 3500/100 000 inhabitants. A few weeks earlier, number of persons tested for SARS-CoV-2 in the French population reached 14 000/100 000 inhabitants before declining as the incidence continued to rise [3].The use of self-testing increased in this period. It is possible that some persons did not report self-testing, despite only serological tests being excluded during data collection. Accordingly, we cannot exclude the possibility that the number of people tested was underestimated in our analyses. Finally, over this last period of the study, a higher proportion of people were difficult to reach by phone; these persons may have had characteristics that were not taken into account in the determinants used in the multivariate analyses. Furthermore, caution is advised when interpreting the results of the analyses of this last period, as a smaller number of persons were interviewed.

Characteristics of the various diagnostic tests available for COVID-19 have been widely described [19] and the impact of their imperfect penfoCOVID France COVID-19: kerformance analysed [20]. However, modelling suggests that the effectiveness of controlling the pandemic depends largely on testing, the frequency of testing, and the speed of reporting [19, 21]. In the context of control, correctly describing persons not tested is fundamental in order to ensure the disease burden is not underestimated or the pandemic’s dynamics misinterpreted.

Diagnostic testing varied according to the characteristics of symptomatic persons: women were tested more frequently (vs. men), as were employed persons (vs. unemployed), persons living with others (vs. 1-person households), healthcare professionals (vs. general population). These results suggest that the decision to be tested was associated with a greater risk of exposure to COVID-19 linked to a greater number of social contacts and to more frequent exposure (for healthcare professionals) to persons who had COVID-19.

Following a positive test, people were obliged to self-isolate and were given sick leave; these two consequences may also have influenced testing practices. It is also true that the decision to perform a test may be altruistic in nature, linked with a desire to limit the risk of transmission.

Almost 10% of participants reported testing positive for SARS-CoV-2 at least once in the previous 12 months. This proportion varied according to the date of the interview and increased over time. This result was expected as the development of the pandemic and the multiplication of pandemic waves over time led to a higher probability that people interviewed later were infected. Retired and ‘other inactive’ persons, as well as persons older than 65 years were less likely to report being infected than employed persons and younger adults. This result may be explained by fewer social interactions for these populations, but also by better adherence to preventive measures and higher COVID-19 vaccination coverage in persons over 64 years (as of 30 December 2021, 71% of persons over 64 years had received a booster dose in France vs. 23% for 18–24 years) [22]. Furthermore, higher infection rates were reported in persons living in large agglomerations and in the Paris area, which is coherent with increased transmission in areas with high population density.

A majority of infected persons knew where they were contaminated (65%). The majority (50.7%) of contaminations occurred in homes (own, family or friend’s). The risk of infection increased with the number of people living in a household, which reflects findings in previous studies [10, 23]. Contamination in homes was more probable in persons who were more likely to stay at home (i.e., retired, unemployed and ‘other inactive’ persons) in comparison with employed persons. Meta-analysis showed that highest secondary-attack rates (SAR) were observed in households, that they increased with the time spent inside a home, and increased over time depending on the variants involved (wild-type: 19%, Alpha: 36%, Delta: 30%, Omicron: 43%) [14, 24]. The probability for contamination at home may have increased because of lockdowns, curfews, recommendations that persons in contact with a COVID-19 case stay at home for one week (for all contacts up to January 2022 in France), and teleworking, which was encouraged during the crisis [25, 26]. However, one case-control study showed that part-time and full-time teleworking were protective against infection in comparison to in-situ office work [10].

The high contamination rates in homes raises the question of how to limit infection in households where sustained family daily contact occurs. Adherence to prevention control measures - including hand, social distancing and wearing masks - are difficult or impossible to implement in a family setting over extended periods of time, especially when young children are present. Studies have shown that SAR related to contacts with family and friends were higher than those for occasional contacts [14]. Because of the transmission characteristics of the virus, this result raises the question of the ability to reduce transmission in households with a large number of inhabitants, especially children.

Our study shows the high probability of contamination in the workplace (29.1% of persons who tested positive), and generally in unventilated closed environments.

A major result is the high rate of healthcare structure in reported work-based contamination (32%). This is to be expected, since healthcare professionals were at a greater risk of infection (PRa: 1.5 [1.3–1.7]) [6, 11, 27]. Healthcare structures, whether frequented in a professional or patient/visitor context, were the second most reported places of contamination (13.9% [11.9–16.1]) after households. This result highlights the burden of healthcare-associated infections and the importance of implementing preventive measures in healthcare structures for professionals, patients, and visitors. Supporting this recommendation, a recent meta-analysis did not identify differences in the pooled SAR between patient and healthcare staff contacts [14]. It must be noted that visits to healthcare facilities has not always been associated with a greater risk of COVID-19 contamination [11]. Control measures and recommendations - including hand washing, wearing a mask, physical distancing, vaccination and confirmed negative testing for visitors - should be insisted on [26, 28]. COVID-19 vaccination is mandatory in France for all healthcare professionals and other personnel working in healthcare facilities. A lack of access to personal protective equipment especially during the first months of the pandemic may have contributed to increased contamination in these populations. With regard to different infection rates observed for different professional categories [6], awareness-raising actions on control measures could be useful, in particular for the least-qualified personnel working in these structures.

The ComCor study identified a greater risk of contamination in bars and restaurants [10]. In our study, these locations accounted for 3.8% of reported places of contamination, rising to 9.0% when frequenting eating places including restaurants, cafeterias and proximity to a coffee machine in the professional context were included. Eating places therefore constituted the third most frequently cited place of contamination by those infected. Control measures are difficult to implement in these structures, as they are places where social interactions are important and customers cannot wear a mask during a meal.

In studies elsewhere, no excess risk was reported for visiting shops, cultural or religious places, or for the use of public transport (except for carpooling) [10]. In our study, we were not able to make any conclusions in terms of excess risk because of the absence of comparison with controls. Shopping or going to a shop accounted for 5.4% of the reported places of contamination and public transport 4.9%. Non-collective transport (car, ambulance, taxi), places of worship and cultural places were rarely mentioned in our study (0.7%, 0.5% and 0.4%, respectively, of all reported places of contamination).

The principal strength of this COVID-19 national study is that highlights the most likely places of contamination at the national level in France, based on reports from infected persons. Our results make it possible to better prioritize actions to limit the circulation of the SARS-COV-2 virus.

The study’s limitations are mostly related to self-reporting, which may lead to over- or under-estimations. The suspected case definition of COVID-19 given to participants was deliberately very broad in accordance with the recommendations for carrying out diagnostic tests. With the objective to identify the cases with a high sensitivity, realization of a diagnostic test was recommended for all suspicious symptoms, without a reduced list of clinical symptoms and with or without contact with a known case of COVID-19 case. Furthermore, memory bias cannot be excluded due to the 12-months window period considered. Moreover, we did not describe adherence to prevention measures, which limits interpretation of the results on places of contamination. In addition, the fact that only fluent French speakers were included means that the disease burden at a national level may have been underestimated. Finally, people who were unable to answer the telephone and elderly residents living in nursing homes, were not included in this study.

## Conclusions

The results of this study are consistent with current knowledge about the modes of transmission of SARS-CoV-2 and with the various measures recommended during the related COVID-19 health crisis. The use of diagnostic testing was decisive for the implementation of control measures and must be performed more systematically for the objective to control the outbreak. Communication actions on the benefits of testing should be highlighted, especially to men, unemployed persons, and those living alone. In order to limit viral spread, preventive actions should preferentially target contamination in households, eating places, and healthcare structures. However, adherence to prevention measures is difficult to achieve in the first two of these settings.

## Electronic supplementary material

Below is the link to the electronic supplementary material.

Supplementary Material 1

## Data Availability

The datasets used and/or analysed during the current study are available from the corresponding author upon reasonable request.

## Abbreviations

PR: Prevalence ratio
PRa: Adjusted prevalence ratio
