# Supplementary material for: SARS-CoV-2 testing, infection and places of contamination in France, a national cross-sectional study, December 2021
Source: BMC Infect Dis. 2023 May 3;23:279. doi: 10.1186/s12879-023-08257-1 (PMC10155649; doi:10.1186/s12879-023-08257-1)
Supplement: Supplementary file 1 — Supplementary Material 1 [file 12879_2023_8257_MOESM1_ESM.docx]

Supplementary data.

Figure 1. Evolutions of numbers of persons tested for SARS-CoV-2 (/ 100 000 inhabitants) and of Covid-19 incidence rate (/ 100 000 inhabitants), from week 20-2020 to week 53-2021, general population, France (Santé publique France)


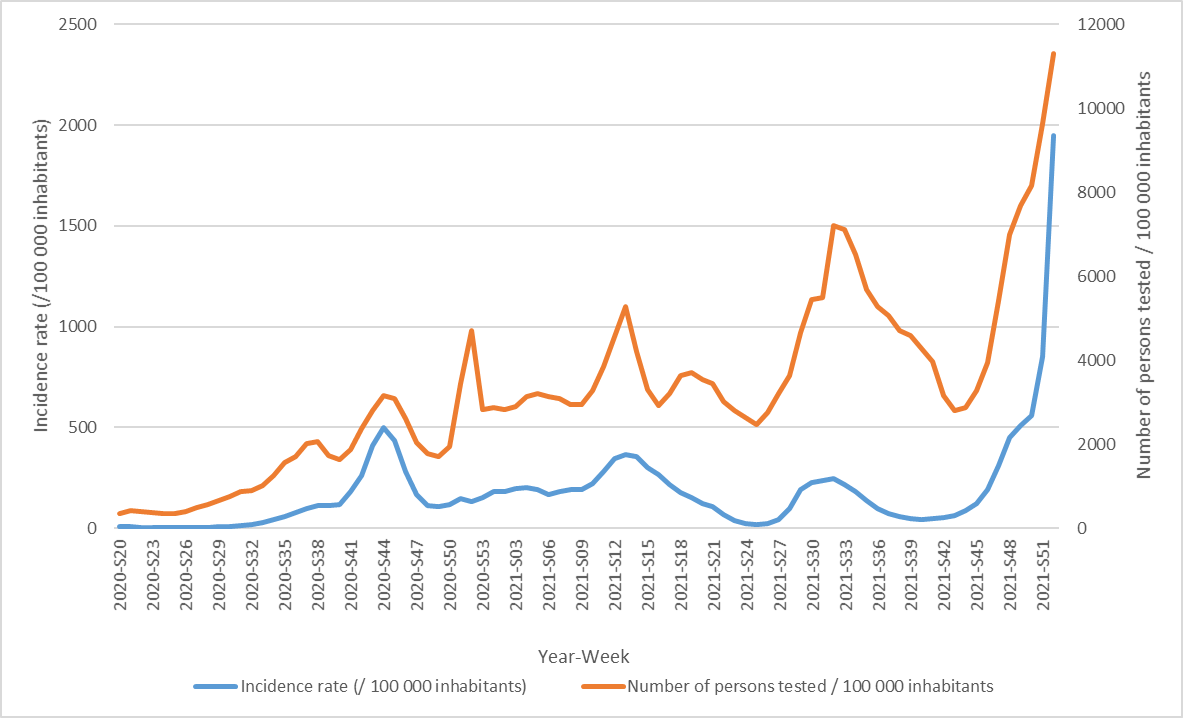


Data took into account public holidays

Figure 2. Evolution of positivity rates for persons tested for SARS-CoV-2 (%), from week 20-2020 to week 53-2021, general population, France (Santé publique France)

Data took into account public holidays
